# The Association of Economic Outcome and Geriatric Syndromes among Older Adults with Transcatheter Aortic Valve Replacement (TAVR)

**DOI:** 10.36469/jheor.2020.17423

**Published:** 2020-10-05

**Authors:** Min Ji Kwak, Rafia Rasu, Robert O. Morgan, Jessica Lee, Nahid J. Rianon, Holly M. Holmes, Abhijeet Dhoble, Dae Hyun Kim

**Affiliations:** 1 University of Texas Health Science Center at Houston, McGovern Medical School, Houston, TX; 2 University of North Texas Health Science Center, Fort Worth, TX; 3 University of Texas Health Science Center at Houston, School of Public Health, Houston, TX; 4 Beth Israel Deaconess Medical Center, Harvard University, Boston, MA

**Keywords:** transcatheter aortic valve replacement, hospital costs, length of stay, delirium, frailty, dementia

## Abstract

**Background:**

The association of geriatric syndromes and economic outcomes among patients who are undergoing transcatheter aortic valve replacement (TAVR) remains unknown.

**Methods and Results:**

A retrospective observational study using the National Inpatient Sample (NIS) from 2011 to 2014 was conducted with 7078 patients who were 65 years or older and underwent TAVR. The average hospital cost was US$58 703 (± SD 29 777) and length of stay (LOS) was 8.1 days (±7.20). The rates of delirium, dementia, and frailty were 8.0%, 6.1%, and 10.5%, respectively. From a multivariable generalized linear regression, delirium increased the cost by 31.5% (95% CI 25.41~37.92) and LOS by 70.3% (95% CI 60.20~83.38). Frailty increased the cost by 7.4% (95% CI 3.44~11.53) and the LOS by 22.6% (95% CI 15.15~30.55). Dementia had no significant association with either outcome. When the interactions of the geriatric syndromes were tested for association with the outcomes, delirium in the absence of dementia but presence of frailty showed the strongest association with cost (increase by 45.1%, 95% CI 26.45~66.45), and delirium in the absence of both dementia and frailty showed the strongest association with LOS (increase by 74.5%, 95% CI 62.71~87.13). When the average hospital cost and LOS were predicted using the model with interaction terms, patients with delirium and frailty (but without dementia) had the highest value (total hospital cost US$86 503 and LOS 14.9 days).

**Conclusion:**

Among TAVR patients, delirium was significantly associated with increased hospital cost and LOS, and the association was significantly higher in the absence of dementia. The results of this study will be a great asset for health care providers and administrators in planning for efficient care strategy to lower health care expenditure in the hospital for older adults who underwent TAVR.

## INTRODUCTION

Transcatheter aortic valve replacement (TAVR) is indicated as an alternative option to surgical aortic valve replacement for the treatment of symptomatic severe aortic stenosis among patients who are at high risk for surgery. Since the US Food and Drug Administration approved TAVR in 2011, indications for TAVR have expanded, and it is now considered to be standard for frail older adults with severe aortic stenosis.^1^,^2^ Patients who are undergoing TAVR tend to be older and frail, and are subsequently at higher risk of developing delirium.^3^–^5^ Delirium after a cardiac procedure such as TAVR is associated with adverse clinical and economic outcomes, including higher perioperative mortality, longer hospital stay, and higher total hospital cost.^5^–^10^ Since TAVR is less invasive than surgical aortic valve replacement, the incidence of delirium after TAVR is lower, but delirium still affects up to 44% of the patients undergoing TAVR.^5^,^7^,^11^

Older adults with a high risk of delirium tend to have coexisting geriatric syndromes such as dementia, frailty, or a combination of more than one. However, studies to assess the association of such geriatric syndromes or their combinations on health care cost and utilization among patients who underwent TAVR are lacking. As the aging population is growing in society, the number of patients who are undergoing TAVR will increase, and so will be the number of patients who will have an adverse effect from various geriatric syndromes. Understanding the magnitude of the association of geriatric syndromes on economic outcome among TAVR patients will assist us in establishing a cost-effective strategy to optimize the clinical benefit with less cost. Therefore, we conducted a study to assess the extent of the association of geriatric syndromes and their combinations on total hospital cost and length of stay (LOS) among patients who underwent TAVR in the United States from 2011 to 2014.

## MATERIALS AND METHODS

### Data Source and Study Design

We conducted a retrospective observational study using the National Inpatient Sample (NIS) data from the Agency for Healthcare Research and Quality (AHRQ), from 2011 to 2014. The NIS data set is an administrative data set developed by AHRQ and it is the largest all-payer inpatient database in the United States. It is designed to approximate a 20% stratified sample of all discharges and represent all nonfederal inpatient admissions in the United States.^12^ Patients were 65 years or older and underwent TAVR, as identified by the International Classification of Diseases, Ninth Revision, Clinical Modification (ICD-9-CM) procedure codes 35.05 and 35.06. Exclusion criteria included patients with a preexisting diagnosis of congenital heart disease or infective endocarditis, or who underwent other cardiac procedures. Patients with missing values for demographic findings and outcome information were also excluded. Relevant ICD-9-CM codes that were used for cohort selection are listed in Table S1. The current study received exempt status by the Institutional Review Board of the University of Texas Health Science Center at Houston.

### Measurements

Geriatric syndromes (delirium, dementia, and frailty) were identified using relevant ICD-9-CM codes. Delirium was identified using the ICD-9-CM codes from the literature except for the codes for “drug-induced persisting dementia” and “dementia with depressed mood or delusional features” (292.82, 290.12, 290.13, 290.43, 290.2, and 290.42), which were used to identify dementia.^13^,^14^ To increase the sensitivity of detecting delirium from the administrative data, information on antipsychotics use is recommended, but NIS does not provide it. Therefore we used only ICD-9-CM codes that were used in the same literature.^14^ Dementia and frailty were identified based on the ICD-9-CM codes that were previously used in the literature with the addition of relevant codes (i.e., 780.71, 780.72, and 780.79 for frailty identification).^15^,^16^ For frailty identification, the original article used both ICD-9-CM codes and the Healthcare Common Procedure Coding System (HCPCS) codes combined with subsequent scoring systems, but we only used the list of ICD-9 codes to identify frailty since NIS does not provide HCPCS codes. If there were any frailty-related ICD-9 codes, then it was considered to have frailty. We used both ICD-9-CM codes and Clinical Classification Software (CCS) codes for identifying other comorbidities for Charlson Comorbidity Index (CCI), atrial fibrillation, coronary artery disease, cerebrovascular disease, congestive heart failure, hypertension, chronic obstructive pulmonary disease, peripheral artery disease, cancer, diabetes, liver disease, and other valvular diseases. CCS codes, provided by the AHRQ, are a tool for classifying the patient’s diagnosis using ICD-9-CM codes.^17^ ICD-9-CM codes and CCS codes that were used to identify geriatric syndromes and comorbidities are listed in Tables S1 and S2. NIS contains total hospital charge information, and the total hospital cost can be estimated from the total charge using the cost-to-charge ratio that was provided by AHRQ.^12^ The cost-to-charge ratio is hospital specific, based on all-payer inpatient cost for nearly every hospital in the database. The cost information that was used to estimate the ratio was obtained by the Centers for Medicare & Medicaid Services from each hospital. All cost was adjusted to 2014 US dollars using the consumer price index. LOS is provided by the data set, by subtracting the admission date from the discharge date.

### Statistical Analysis

All the statistical analyses were conducted using Stata 14.2 (College Station, TX). Descriptive statistics for all study variables were conducted first, followed by multivariable linear regression analyses. A two-sided *P* value < 0.05 was considered statistically significant. We applied weight to produce national estimates as recommended by AHRQ (variable DISWT). After we confirmed that there were not excessive zero values, we used multivariable generalized linear regression with gamma family and log link to estimate the association of each geriatric syndrome (delirium, dementia, and frailty) on total hospital cost and Poisson family on LOS.^18^–^20^ Covariates for adjustment included age (65–79, 80–89, or >90 years), sex, race (White, Black, Hispanic, Asian or Pacific Islander, Native American, or other), insurance (Medicare/Medicaid, private insurance, self-pay, or others), admission type (elective or emergent), urban or rural location, and comorbidities (see previous section) that were selected based on previously published literature and clinical judgment.^21^–^23^ CCI was not included in the regression model due to high multicollinearity.

We then tried to assess the association of the interaction terms of geriatric syndromes with the outcomes. Finally, we obtained the average total hospital cost and LOS for the combinations of the geriatric syndromes using the “margins” command in Stata.

### Sensitivity Analysis

We conducted a one-way sensitivity analysis to address the possibility of underreporting of the geriatric syndrome. Since NIS is a discharge data set in which the diagnosis codes were collected at the time of discharge, identification of geriatric syndrome based on discharge diagnosis has an inherent limitation of measurement errors. Furthermore, it does not provide information of HCPCS codes or antipsychotics use to increase the sensitivity of identifying frailty and delirium per the algorithm by Kim et al.^14^,^15^ To overcome this limitation, we constructed a new data set by resampling the original data to approximate the prevalence of geriatric syndromes from the literature.^5^,^7^,^11^,^24^,^25^ With multiple resampling and repetition,^26^ we were able to create a simulated data set with a rate of delirium among TAVR patients as 43%, 34% for dementia, and 47% for frailty. With the new simulated data set, we repeated the process of obtaining the final model to estimate the association of combinations of geriatric syndromes and the outcome.

## RESULTS

**Figure 1:**
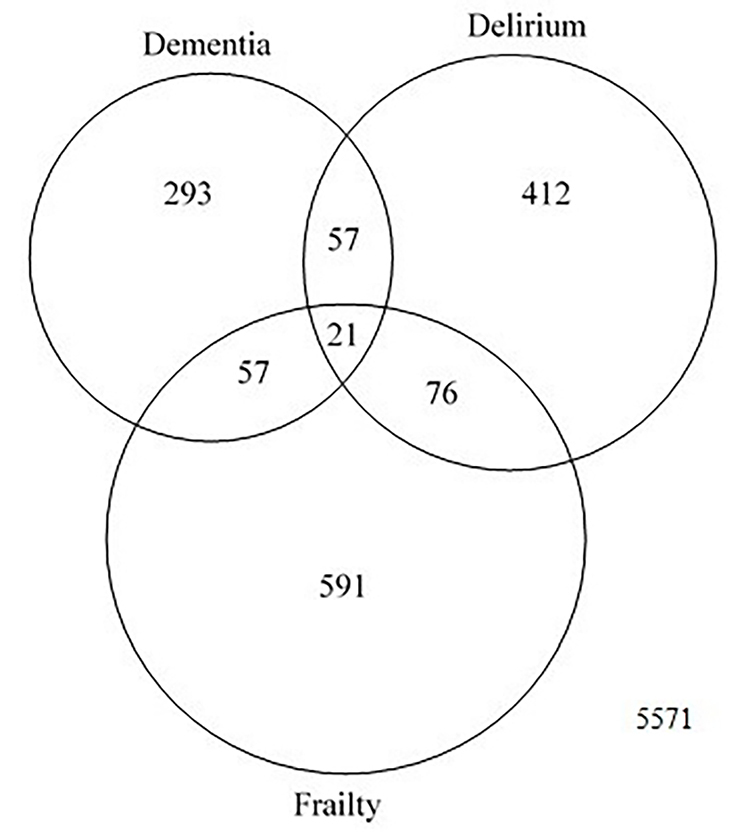
Number of Patients with Geriatric Syndromes who Underwent TAVR

From the total 29 511 883 patients from NIS 2011–2014 data set, we identified 7078 patients who met the inclusion and exclusion criteria. The study population had 55.3% in age 80–89 years, 48.2% female, 87.5% White, and 93.6% with Medicare or Medicaid. Most of the procedures were elective (76.1%). The average CCI was 2.62 (SD ±1.61). Eight percent of the patients had delirium, 10.5% had diagnoses related to frailty, and 6.1% had diagnoses of dementia (see Table 1). Figure 1 shows the number of patients with each geriatric syndrome and their combinations. Among 7078 patients, 293 people had dementia only, 412 had delirium only, and 591 were diagnosed with frailty. Fifty-seven people had both dementia and delirium, another 57 had both dementia and frailty, and 76 had delirium and frailty. Twenty-one patients had all the geriatric syndromes: delirium, dementia, and frailty.

When each geriatric syndrome was assessed separately with a multivariable regression model adjusting other covariates, delirium showed the largest association on the total hospital cost (31.5% increase in total hospital cost compared to patients without delirium, 95% CI 25.41~37.92). Frailty also had a significant association, with an increased cost of 7.4% from those without frailty (95% CI 3.44~11.53), but dementia did not show a significant association. Similarly, the LOS showed significant association with delirium (increase of 70.3%, 95% CI 60.20~80.99) and frailty (increase of 22.6%, 95% CI 15.15~30.55), but not by dementia (see Table 2). The result of the multivariable regression with the coefficients and incidence rate ratio for all the covariates are reported in Table S3.

From the model with the interaction terms of the three geriatric syndromes, delirium had the biggest association with cost in the absence of dementia but the presence of frailty, increasing the cost by 45.1% (95% CI 26.45~66.45). Dementia was significantly associated with lower hospital cost when it was present with delirium but without frailty (decreased the cost by 16.53%, 95% CI −25.89~−5.97), and when it was present with both delirium and frailty (decreased the cost by 30.17%, 95% CI −41.51~−16.61). Frailty was associated with a higher cost when it was present in the absence of both dementia and delirium (increased the cost by 4.9%, 95% CI 0.92~9.01). Delirium consistently showed significant association with longer LOS in the hospital in either condition; with presence or absence of dementia and frailty. Delirium showed the biggest association with the LOS when it was present in the absence of both dementia and frailty, increasing the LOS by 74.5% (95% CI 62.71~87.13), followed by the condition when dementia was present but frailty was absent (increasing the LOS by 66.1% with 95% CI 39.50~97.85). When dementia was present but frailty was absent, delirium increased the LOS by 37.1% (95% CI 13.70~65.20), and when both dementia and frailty were present, delirium increased the LOS by 36.2% (95% CI 1.78~82.30). Dementia was associated with shorter LOS in the hospital when delirium was present but frailty was absent (decrease by 21.7% with 95% CI −34.73~−6.03). Like hospital cost, frailty only was associated with longer LOS when it was present in the absence of both dementia and delirium (increase by 20.2% with 95% CI 12.08~28.84).

**Figure 2:**
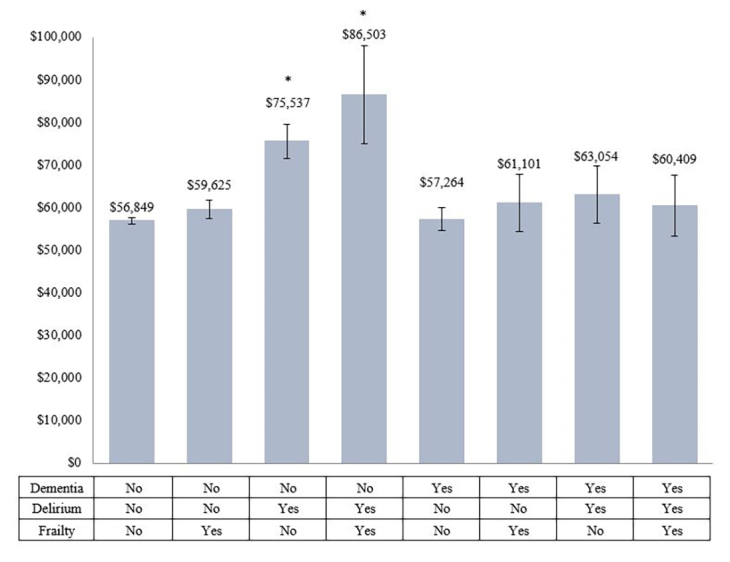
Predicted Average Total Hospital Cost of Each Condition from the Final Regression Model with Interaction Terms * Indicates statistically significant difference from the condition without any geriatric syndromes.

**Figure 3:**
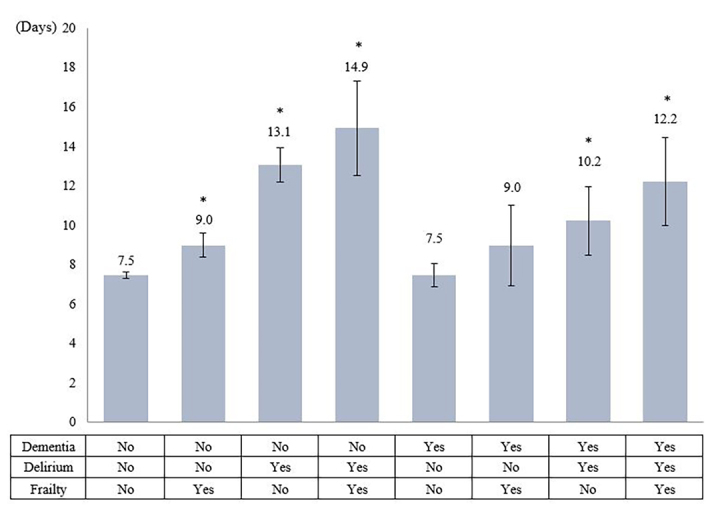
Predicted Average LOS for Each Condition from the Final Regression Model with Interaction Terms * Indicates statistically significant difference from the condition without any geriatric syndromes.

From the final model with the interaction terms, the average total hospital cost was the highest among those who had delirium and frailty, but no dementia (US$86 503 with 95% CI 75 052.2~97 953.6), followed by those who had delirium only (US$75 537 with 95% CI 71 468.7~79 604.9). Patients without any geriatric syndromes showed the lowest cost, US$56 849 (95% CI 56 162.4~57 534.8) (Figure 2). The average LOS was the highest among those with delirium and frailty (14.9 days with 95% CI 12.52~17.34), followed by those with only delirium (13.1 days with 95% CI 12.19~13.91). Patients without any geriatric syndromes had LOS of 7.48 (95% CI 7.32~7.64), but patients with only dementia had slightly shorter LOS (7.46) with 95% CI 6.87~8.04, without statistically significant difference (Figure 3).

Other than the geriatric syndromes and their interactions, significant association with higher cost and longer LOS was shown for female sex, urban location, atrial fibrillation, cerebrovascular disease, congestive heart failure, chronic kidney disease, or liver disease (see Table S4).

The result of the sensitivity analysis confirmed that delirium showed the biggest association with the cost, increasing by 25.7% (95% CI 19.03~32.56). However, dementia lowered the cost by 27.47% (95% CI −21.86~−12.83) and frailty did not show significant association. The association with the LOS showed a similar pattern: delirium also showed significant association with longer LOS, increasing by 48.7% (95% CI 36.95~61.56), dementia shortened the LOS by 25.8% (95% CI −31.93~−19.06), and frailty did not show significant association. When the interaction terms were tested, it was consistently shown that delirium had a significant association with higher cost with any combination of geriatric syndromes—with presence or absence of dementia or frailty—increasing the cost by 11.6%~37.9%, and longer LOS, increasing the LOS by 44.9%~118.9%. Dementia or frailty had various associations according to the combinations of geriatric syndromes in a similar pattern with the original data set.

## DISCUSSION

The current study evaluated the association of delirium, dementia, or frailty, and their combinations on total hospital cost and LOS among patients who underwent TAVR in the United States from 2011 to 2014. Our study has a strength of assessing the association of not only a single geriatric syndrome (delirium, dementia, and frailty) but also the combinations of them on economic outcomes using a nationally representative data set. The result showed that delirium and frailty had a significant association with the hospital cost or the LOS, and delirium showed the strongest association. When it comes to the effect of combinations of the geriatric syndromes, we suggest that delirium in the absence of dementia but the presence of frailty had the strongest effect on cost, and delirium in the absence of both dementia and frailty had the biggest association with the LOS. However, in the presence of dementia, regardless of the presence of frailty, delirium did not have a significant association with the cost but did on the LOS. Frailty only impacted the cost or LOS when both dementia and delirium were absent. When the average hospital cost and LOS were predicted using the final regression model, patients with delirium and frailty but without dementia had the highest value (total hospital cost US$86 503 and LOS 14.9 days).

The results from the sensitivity analysis using a simulated data set reflecting the higher rate of those geriatric syndromes also confirmed that delirium had the strongest effect on cost. Our findings are consistent with the results of previous studies, showing that delirium or frailty increases the hospital cost or LOS.^9^,^10^,^27^ While frailty is a rather underlying condition, delirium develops during the hospitalization; considering that it poses the most substantial impact, it resonates with the importance of the effort to prevent delirium among patients who are undergoing TAVR.

Interestingly, dementia itself did not show a significant association with the hospital cost and LOS. Furthermore, in the presence of delirium, it was rather associated with lower hospital cost and LOS compared to those without it. This could be due to the LOS among patients who developed delirium without underlying dementia requiring transfer to other facilities compared to those who already had dementia and had been in a facility before admission. Since dementia is the most common geriatric syndrome among nursing home residences, patients with a diagnosis of dementia might have been admitted from nursing homes.^28^ Therefore, their discharge planning might have been quicker than for the patients without a prior diagnosis of dementia but who had developed delirium. When we estimated the average daily hospital cost for patients with the multivariable regression with interaction terms, adjusting patients’ demographic and clinical factors, we saw that those who had delirium without dementia showed lower daily hospital costs (US$7199 with 95% CI 6805.7~7592.4) than those who developed delirium with dementia (US$7765 with 95% CI 6511.1~9019.2). This finding may be a reflection of the days in the hospital without further treatment or workups waiting for the transfer to a facility. If we had the information about the source of admission, we would have been able to test this hypothesis, but NIS does not provide information regarding admission sources from 2012. They collected information about the admission source in 2011, but more than 50% of the observations were missing. Therefore, we were not able to conduct analysis accounting for the source of admission. This is a certainly an unknown area, and further prospective research will be necessary to estimate the exact impact of dementia combined with delirium on utilization of health care resources.

The current study has limitations. First, this study is a retrospective study using a discharge data set, which has potential measurement errors. If the provider does not register the patient’s diagnosis related to coexisting dementia or frailty, those conditions may be underreported. For example, HCPCS codes or medication use for more accurate identification of frailty or delirium were not available from the NIS data set. Therefore, we conducted a rigorous sensitivity analysis by resampling the original data to construct a new simulated data set reflecting the prevalence of geriatric syndromes that were obtained from the literature. Since there were overlaps among the geriatric syndromes, and there was a lack of information regarding the prevalence of the overlapping conditions, we were only able to approximate the prevalence from the literature. For example, the literature reported that the rate of delirium up to 44%, dementia 32%, and frailty 49%, 5,7,11,24,25 but we were able to construct the data set with delirium at 43%, dementia 34%, and frailty 47%. Nevertheless, the importance of further study using prospective data with more accurate clinical information regarding geriatric syndromes to assess the interactions on economic outcome cannot be ignored in order to validate the result of the current retrospective study. Furthermore, data from 2011 to 2014 may not reflect the more recent years’ practice, considering that the management of TAVR has been rapidly improving. However, because AHRQ changed the diagnosis coding system in 2015, we were not able to combine more recent data. When more recent data become available, future studies will be warranted.

Another limitation of the study is that the economic outcome was assessed from the hospital’s perspective. We did not assess the cost from a societal perspective, payer’s perspective, or patient perspective. Care for patients who were discharged to a facility also spend a significant amount of health care expenditure from societal or payer’s perspective. NIS only provides information about total hospital costs, but a further in-depth study is necessary to assess the actual economic impact of various geriatric conditions among TAVR patients.

Despite the limitations, we believe that the result of our study holds significant value. To the best of our knowledge, this is the first study assessing to what extent geriatric syndromes influence on hospital cost and LOS among patients who underwent TAVR, using a nationally representative data set. Previous studies have evaluated the effect of delirium on health care resources utilization among patients with TAVR, but not other geriatric syndromes (frailty and dementia or combinations of those three geriatric syndromes).^9^,^10^ Our results showed that geriatric syndromes, especially delirium, play a substantial role in increasing health care expenditure for patients with TAVR.

With rising health care expenditures, there has been significant pressure on providers to practice a better quality of care with less cost. Therefore, it is essential to know the exact magnitude of health care cost required to take care of patients with certain conditions in order to plan a more efficient care strategy and policy implementation, consequently lowering the hospital cost according to the patients’ preexisting geriatric syndrome. Additionally, hospital administrators or policymakers can make a more accurate financial plan to reallocate their resources, preventing delirium in order to save on costs. Accurate information regarding economic outcome according to the patients’ various medical conditions will be a great asset to perform a future economic evaluation and identify the most efficient policy. The result of the current study will be a foundation for a better quality of care for older adults with various geriatric syndromes and who undergo TAVR procedure.

## Supplementary Information



## Figures and Tables

**Table 1:** Baseline Characteristics of the Patients who Underwent TAVR from 2011 to 2014

Variables		Patients with TAVR (N = 7078)
Age group (N, [%])	65–79	2050 (28.96%)
	80–89	3915 (55.31%)
	≥90	1113 (15.72%)
Female (N, [%])		3414 (48.23%)
Race (N, [%])	White	6193 (87.50%)
	Black	259 (3.66%)
	Hispanic	247 (3.49%)
	Asian or Pacific Islander	76 (1.07%)
	Native American	13 (0.18%)
	Other	290 (4.10%)
Insurance (N, [%])	Medicare/Medicaid	6625 (93.60%)
	Private	347 (4.90%)
	Self-pay	26 (0.37%)
	Other	80 (1.13%)
Elective admission (N, [%])		5381 (76.10%)
Charlson’s Comorbidity Index (Mean±SD)		2.62±1.61
Atrial fibrillation (N, [%])		3258 (53.97%)
Coronary arterial disease (N, [%])		5134 (72.53%)
Cerebrovascular disease (N, [%])		759 (10.72%)
Congestive heart failure (N, [%])		5254 (74.23%)
Hypertension (N, [%])		5836 (82.45 %)
Chronic obstructive pulmonary disease (N, [%])		1958 (27.66 %)
Peripheral arterial disease (N, [%])		1660 (23.45 %)
Chronic kidney disease (N, [%])		2683 (37.91%)
Cancer (N, [%])		1609 (22.73 %)
Diabetes (N, [%])		2697 (38.10 %)
Liver disease (N, [%])		253 (3.57 %)
Other valvular disease (N, [%])		1165 (16.46 %)
Urban area (N, [%])		5867 (83.33%)
Delirium (N, [%])		564 (8.0%)
Dementia (N, [%])		428 (6.1%)
Frailty (N, [%])		745 (10.5%)

Abbreviations: TAVR, transcatheter aortic valve replacement.

**Table 2:** Changes of Total Hospital Cost and LOS with Geriatric Syndromes

Without Interaction Terms				
Delirium	**Total Hospital Cost**		**LOS**	

	**Changes in % (95% CI)**		**Changes in % (95% CI)**	

	↑31.5% a	(↑25.41~↑37.92)	↑70.3% a	(↑60.20~↑80.99)

Dementia	↑0.1%	(↓0.04~↑4.28)	↑2.1%	(↓4.90~↑9.60)

Frailty	↑7.4% a	(↑3.44~↑11.53)	↑22.6% a	(↑15.15~↑30.55)

**With Interaction Terms**				

**Change**	**Total Hospital Cost**		**LOS**	

	**Changes in % (95% CI)**		**Changes in % (95% CI)**	
	
From delirium in the absence of both dementia and frailty	↑32.9%^a^	(↑25.72~↑40.43)	↑74.5% a	(↑62.71~↑87.13)

From delirium in the presence of dementia but absence of frailty	↑10.1%	(↓1.96~↑23.67)	↑37.1% a	(↑13.70~↑65.20)

From delirium in the absence of dementia but presence of frailty	↑45.1% a	(↑26.45~↑66.45)	↑66.1% a	(↑39.50~↑97.85)

From delirium in the presence of both dementia and frailty	↓1.13%	(↓15.84~↑16.15)	↑36.2% a	(↑1.78~↑82.30)

From dementia in the absence of both delirium and frailty	↑0.7%	(↓4.10~↑5.81)	↓0.3%	(↓8.02~↑8.10)

From dementia in the presence of delirium but absence of frailty	↓16.53% a	(↓25.89~↓5.97)	↓21.7% a	(↓34.73~↓6.03)

From dementia in the absence of delirium but presence of frailty	↑2.5%	(↓8.72~↑15.04)	↓0.2%	(↓21.22~↑26.36)

From dementia in the presence of both delirium and frailty	↓30.17% a	(↓41.51~↓16.61)	↓18.2%	(↓35.94~↑4.46)

From frailty in the absence of both dementia and delirium	↑4.9% a	(↑0.92~↑9.01)	↑20.2% a	(↑12.08~↑28.84)

From frailty in the presence of dementia but absence of delirium	↑6.7%	(↓5.30~↑20.22)	↑20.3%	(↓5.42~↑52.88)

From frailty in the presence of both dementia and delirium	↓4.2%	(↓18.3~↑12.31)	↑19.5%	(↓6.95~↑53.51)

From frailty in the absence of dementia but presence of delirium	↑14.5%	(↓0.70~↑32.06)	↑14.4%	(↓3.83~↑36.12)

a Indicates statistically significant association.
