# Supplementary material for: The Association of Economic Outcome and Geriatric Syndromes among Older Adults with Transcatheter Aortic Valve Replacement (TAVR)
Source: J Health Econ Outcomes Res. 2020 Oct 5;7(2):175–81. doi: 10.36469/jheor.2020.17423 (PMC7549540; doi:10.36469/jheor.2020.17423)
Supplement: Supplementary file 1 [file jheor-7-2-17423-s01.pdf]

Journal of Health Economics and Outcomes Research

Supplementary Online Material

Kwak MJ, Rasu R, Morgan R, et al.. The association of economic outcome and geriatric syndromes among older adults with transcatheter aortic valve replacement (TAVR). *JHEOR*. 2020;7(2):175-181.
doi:10.36469/jheor.2020.17423

Table S1. ICD-9-CM and CCS Codes Used for Identifying the Condition
Table S2. ICD-9-CM Codes Used to Identify Charlson's Comorbidity Index
Table S3. Association of Each Geriatric Condition with Total Hospital Cost and LOS
Table S4. Association of Interaction Terms of Geriatric Conditions with Total Hospital Cost and LOS

This supplementary material has been provided by the authors to give readers additional information about their work.

This is an open-access article distributed under the terms of the Creative Commons Attribution 4.0 International License (CCBY-4.0). View this license's legal deed at http://creativecommons.org/licenses/by/4.0 and legal code at http://creativecommons.org/licenses/by/4.0/legalcode for more information.

Table S1. ICD-9-CM and CCS Codes Used for Identifying the Condition

	ICD-9-CM codes	CCS codes
Congenital disease and infective endocarditis for exclusion	746.9, 421, 421.0, 421.1, 421.9	
Other concurrent cardiologic surgery and procedure for exclusion	35.13, 35.25, 35.26, 35.14, 35.27, 35.28, 35.12, 35.31, 35.32, 35.33, 35.23, 35.24, 36.10, 36.11, 36.12, 36.13, 36.14, 36.15, 36.16, 36.17, 36.18, 36.19	
Transcatheter aortic valve replacement	35.05, 35.06	
Hypertension		98, 99
Coronary arterial disease		100, 101
Diabetes		49, 50
Congestive heart failure		108
Peripheral arterial disease		114
Cerebrovascular disease		109, 110, 111
Chronic pulmonary obstructive disease		127
Cancer		11–45
Chronic kidney disease		158
Atrial fibrillation	427.31	
Liver disease	517.00, 571.10, 571.30, 571.40, 571.41, 571.42, 571.49, 571.50, 571.60, 571.80, 571.90	150, 151
Other valvular disease	394.0, 394.1, 394.2, 394.9, 395.1, 395.2, 395.9, 396.1, 396.3, 396.8, 396.9, 397.0, 397.1, 397.9, 424.0, 424.2, 424.3, 424.90, 424.91, 424.99, 785.2, V42.2, V43.3	
Delirium	293, 293.0, 293.1, 292.81, 290.11, 290.3, 290.3 290.41, 291, 291.0, 293.9, 780.09, 293.81, 293.82, 293.83, 293.84, 293.89, 292.11, 292.12, 292.2, 780.02, 290.8, 290.9, 292, 292.0, 348.3, 348.30, 348.31, 348.39, 349.82, 780.97	
Dementia	290.xx, 294.xx, 331.xx, 291.1, 291.2, 292.82, 292.83	
Frailty	781.2, 783.2, 783.7, 799.4, 799.3, 719.7, V15.88, 780.71, 780.72, 780.79, 728.2, 728.87, 707.0x, 707.2x, 797, 797.0, 797	

Abbreviations: CCS, Clinical Classification Software; ICD-9-CM, International Classification of Diseases, Ninth Revision, Clinical Modification

Table S2. ICD-9-CM Codes Used to Identify Charlson's Comorbidity Index

Charlson's Comorbidity	ICD-9-CM Codes
Myocardial infarction	410, 410.0, 410.1, 410.2, 410.3, 410.4, 410.5, 410.9, 410.00, 410.01, 410.02, 410.10, 410.11, 410.12, 410.20, 410.21, 410.22, 410.30, 410.31, 410.32, 410.40, 410.41, 410.42, 410.50, 410.51, 410.52, 410.60, 410.61, 410.62, 410.70, 410.71, 410.72, 410.80, 410.81, 410.82, 410.90, 410.91, 410.92
Congestive heart failure	428, 428.0, 428.1, 428.20, 428.21, 428.22, 428.23, 428.30, 428.31, 428.32, 428.33, 428.40, 428.41, 428.42, 428.43, 428.9
Peripheral vascular disease	433.90, 441, 433.91, 441.00, 441.01, 441.02, 441.03, 441.1, 441.2, 441.3, 441.4, 441.5, 441.6, 441.7, 441.9, 785.4, V43.4
Cerebrovascular disease	430, 431, 432, 433, 434, 435, 436, 437, 438, 432.0, 432.1, 432.9, 433.00, 433.01, 433.10, 433.11, 433.20, 433.21, 433.30, 433.31, 433.80, 433.81, 433.90, 433.91, 434.00, 434.01, 434.10, 434.11, 434.90, 434.91, 435.0, 435.1, 435.2, 435.3, 435.8, 435.9, 436, 437.0, 437.1, 437.2, 437.3, 437.4, 437.5, 437.6, 437.7, 437.8, 437.9, 438.0, 438.10, 438.11, 438.12, 438.13, 438.14, 438.19, 438.20, 438.21, 438.22, 438.30, 438.31, 438.32, 438.40, 438.41, 438.42, 438.50, 438.51, 438.52, 438.53, 438.6, 438.7, 438.81, 438.82, 438.83, 438.84, 438.85, 438.89, 438.9
Dementia	290, 290.0, 290.10, 290.11, 290.12, 290.13, 290.20, 290.21, 290.3, 290.40, 290.41, 290.42, 290.43, 290.8, 290.9
Chronic pulmonary disease	490, 491, 492, 493, 494, 495, 496, 500, 501, 502, 503, 504, 505, 491.0, 491.1, 491.20, 491.21, 491.22, 491.8, 491.9, 492.0, 492.8, 493.00, 493.01, 493.02, 493.10, 493.11, 493.12, 493.20, 493.21, 493.22, 493.81, 493.82, 493.90, 493.91, 493.92, 494.0, 494.1, 495.0, 495.1, 495.2, 495.3, 495.4, 495.5, 495.6, 495.7, 495.8, 495.9, 496, 500, 501, 502, 503, 504, 505, 506.4
Connective tissue disease	710.0, 710.1, 710.4, 714.0, 714.1, 714.2, 714.81, 725
Peptic ulcer disease	531.00, 531, 532, 533, 534, 531.01, 531.10, 531.11, 531.20, 531.21, 531.30, 531.31, 531.40, 531.41, 531.50, 531.51, 531.60, 531.61, 531.70, 531.71, 531.90, 531.91, 532.00, 532.01, 532.10, 532.11, 532.20, 532.21, 532.30, 532.31, 532.40, 532.41, 532.50, 532.51, 532.60, 532.61, 532.70, 532.71, 532.90, 532.91, 533.00, 533.01, 533.10, 533.11, 533.20, 533.21, 533.30, 533.31, 533.40, 533.41, 533.50, 533.51, 533.60, 533.61, 533.70, 533.71, 533.90, 533.91, 534.00, 534.01, 534.10, 534.11, 534.20, 534.21, 534.30, 534.31, 534.40, 534.41, 534.50, 534.51, 534.60, 534.61, 534.70, 534.71, 534.90, 534.91
Mild liver disease	571.2, 571.40, 571.41, 571.42, 571.49, 571.5, 571.6
Diabetes without complications	250.00, 250.01, 250.02, 250.03, 250.10, 250.11, 250.12, 250.13, 250.20, 250.21, 250.22, 250.23, 250.30, 250.31, 250.32, 250.33, 250.70, 250.71, 250.72, 250.73
Diabetes with complications	250.40, 250.41, 250.42, 250.43, 250.50, 250.51, 250.52, 250.53, 250.60, 250.61, 250.62, 250.63
Paraplegia and hemiplegia	344.1, 342, 342.00, 342.01, 342.02, 342.10, 342.11, 342.12, 342.80, 342.81, 342.82, 342.90, 342.91, 342.92
Renal disease	582.0, 582, 582.1, 582.2, 582.4, 582.81, 582.89, 582.9, 583.0, 583.1, 583.2, 583.4, 583.6, 583.7, 585.1, 585.2, 585, 585.3, 585.4, 585.5, 585.6, 585.9, 586, 588.0, 588.1, 588.81, 588.89, 588, 588.9

Continued

Cancer	140.0, 140.1, 140.3, 140.4, 140.5, 140.6, 140.8, 140.9, 141.0, 141.1, 141.2, 141.3, 141.4, 141.5, 141.6, 141.8, 141.9, 142.0, 142.1, 142.2, 142.8, 142.9, 143.0, 143.1, 143.8, 143.9, 144.0, 144.1, 144.8, 144.9, 145.0, 145.1, 145.2, 145.3, 145.4, 145.5, 145.6, 145.8, 145.9, 146.0, 146.1, 146.2, 146.3, 146.4, 146.5, 146.6, 146.7, 146.8, 146.9, 147.0, 147.1, 147.2, 147.3, 147.8, 147.9, 148.0, 148.1, 148.2, 148.3, 148.8, 148.9, 149.0, 149.1, 149.8, 149.9, 150.0, 150.1, 150.2, 150.3, 150.4, 150.5, 150.8, 150.9, 151.0, 151.1, 151.2, 151.3, 151.4, 151.5, 151.6, 151.8, 151.9, 152.0, 152.1, 152.2, 152.3, 152.8, 152.9, 153.0, 153.1, 153.2, 153.3, 153.4, 153.5, 153.6, 153.7, 153.8, 153.9, 154.0, 154.1, 154.2, 154.3, 154.8, 155.0, 155.1, 155.2, 156.0, 156.1, 156.2, 156.8, 156.9, 157.0, 157.1, 157.2, 157.3, 157.4, 157.8, 157.9, 158.0, 158.8, 158.9, 159.0, 159.1, 159.8, 159.9, 160.0, 160.1, 160.2, 160.3, 160.4, 160.5, 160.8, 160.9, 161.0, 161.1, 161.2, 161.3, 161.8, 161.9, 162.0, 162.2, 162.3, 162.4, 162.5, 162.8, 162.9, 163.0, 163.1, 163.8, 163.9, 164.0, 164.1, 164.2, 164.3, 164.8, 164.9, 165.0, 165.8, 165.9, 170.0, 170.1, 170.2, 170.3, 170.4, 170.5, 170.6, 170.7, 170.8, 170.9, 171.0, 171.2, 171.3, 171.4, 171.5, 171.6, 171.7, 171.8, 171.9, 172.0, 172.1, 172.2, 172.3, 172.4, 172.5, 172.6, 172.7, 172.8, 172.9, 174.0, 174.1, 174.2, 174.3, 174.4, 174.5, 174.6, 174.8, 174.9, 175.0, 175.9, 176.0, 176.1, 176.2, 176.3, 176.4, 176.5, 176.8, 176.9, 179, 180.0, 180.1, 180.8, 180.9, 181, 182.0, 182.1, 182.8, 183.0, 183.2, 183.3, 183.4, 183.5, 183.8, 183.9, 184.0, 184.1, 184.2, 184.3, 184.4, 184.8, 184.9, 185, 186.0, 186.9, 187.1, 187.2, 187.3, 187.4, 187.5, 187.6, 187.7, 187.8, 187.9, 188.0, 188.1, 188.2, 188.3, 188.4, 188.5, 188.6, 188.7, 188.8, 188.9, 189.0, 189.1, 189.2, 189.3, 189.4, 189.8, 189.9, 190.0, 190.1, 190.2, 190.3, 190.4, 190.5, 190.6, 190.7, 190.8, 190.9, 191.0, 191.1, 191.2, 191.3, 191.4, 191.5, 191.6, 191.7, 191.8, 191.9, 192.0, 192.1, 192.2, 192.3, 192.8, 192.9, 193, 194.0, 194.1, 194.3, 194.4, 194.5, 194.6, 194.8, 194.9, 195.0, 195.1, 195.2, 195.3, 195.4, 195.5, 195.8, 200.00, 200.01, 200.02, 200.03, 200.04, 200.05, 200.06, 200.07, 200.08, 200.10, 200.11, 200.12, 200.13, 200.14, 200.15, 200.16, 200.17, 200.18, 200.20, 200.21, 200.22, 200.23, 200.24, 200.25, 200.26, 200.27, 200.28, 200.30, 200.31, 200.32, 200.33, 200.34, 200.35, 200.36, 200.37, 200.38, 200.40, 200.41, 200.42, 200.43, 200.44, 200.45, 200.46, 200.47, 200.48, 200.50, 200.51, 200.52, 200.53, 200.54, 200.55, 200.56, 200.57, 200.58, 200.60, 200.61, 200.62, 200.63, 200.64, 200.65, 200.66, 200.67, 200.68, 200.70, 200.71, 200.72, 200.73, 200.74, 200.75, 200.76, 200.77, 200.78, 200.80, 200.81, 200.82, 200.83, 200.84, 200.85, 200.86, 200.87, 200.88, 201.00, 201.01, 201.02, 201.03, 201.04, 201.05, 201.06, 201.07, 201.08, 201.10, 201.11, 201.12, 201.13, 201.14, 201.15, 201.16, 201.17, 201.18, 201.20, 201.21, 201.22, 201.23, 201.24, 201.25, 201.26, 201.27, 201.28, 201.40, 201.41, 201.42, 201.43, 201.44, 201.45, 201.46, 201.47, 201.48, 201.50, 201.51, 201.52, 201.53, 201.54, 201.55, 201.56, 201.57, 201.58, 201.60, 201.61, 201.62, 201.63, 201.64, 201.65, 201.66, 201.67, 201.68, 201.70, 201.71, 201.72, 201.73, 201.74, 201.75, 201.76, 201.77, 201.78, 201.90, 201.91, 201.92, 201.93, 201.94, 201.95, 201.96, 201.97, 201.98, 202.00, 202.01, 202.02, 202.03, 202.04, 202.05, 202.06, 202.07, 202.08, 202.10, 202.11, 202.12, 202.13, 202.14, 202.15, 202.16, 202.17, 202.18, 202.20, 202.21, 202.22, 202.23, 202.24, 202.25, 202.26, 202.27, 202.28, 202.30, 202.31, 202.32, 202.33, 202.34, 202.35, 202.36, 202.37, 202.38, 202.40, 202.41, 202.42, 202.43, 202.44, 202.45, 202.46, 202.47, 202.48, 202.50, 202.51, 202.52, 202.53, 202.54, 202.55, 202.56, 202.57, 202.58, 202.60, 202.61, 202.62, 202.63, 202.64, 202.65, 202.66, 202.67, 202.68, 202.70, 202.71, 202.72, 202.73, 202.74, 202.75, 202.76, 202.77, 202.78, 202.80, 202.81, 202.82, 202.83, 202.84, 202.85, 202.86, 202.87, 202.88, 202.90, 202.91, 202.92, 202.93, 202.94, 202.95, 202.96, 202.97, 202.98, 203.00, 203.01, 203.02, 203.10, 203.11, 203.12, 203.80, 203.81, 203.82, 204.00, 204.01, 204.02, 204.10, 204.11, 204.12, 204.20, 204.21, 204.22, 204.80, 204.81, 204.82, 204.90, 204.91, 204.92, 205.00, 205.01, 205.02, 205.10, 205.11, 205.12, 205.20, 205.21, 205.22, 205.30, 205.31, 205.32, 205.80, 205.81, 205.82, 205.90, 205.91, 205.92, 206.00, 206.01, 206.02, 206.10, 206.11, 206.12, 206.20, 206.21, 206.22, 206.80, 206.81, 206.82, 206.90, 206.91, 206.92, 207.00, 207.01, 207.02, 207.10, 207.11, 207.12, 207.20, 207.21, 207.22, 207.80, 207.81, 207.82, 208.00, 208.01, 208.02, 208.10, 208.11, 208.12, 208.20, 208.21, 208.22, 208.80, 208.81, 208.82, 208.90, 208.91, 208.92, 140, 141, 142, 143, 144, 145, 146, 147, 148, 149, 150, 151, 152, 153, 154, 155, 156, 157, 158, 159, 160, 161, 162, 163, 164, 165, 166, 167, 168, 169, 170, 171, 172, 174, 175, 176, 177, 178, 179, 180, 181, 182, 183, 184, 185, 186, 187, 188, 189, 190, 191, 192, 193, 194, 195, 200, 201, 202, 203, 204, 205, 206, 207, 208
Moderate or severe liver disease	572.2, 572.3, 572.4, 572.8, 572
Metastatic carcinoma	196, 197, 198, 199, 196.0, 196.1, 196.2, 196.3, 196.5, 196.6, 196.8, 196.9, 197.0, 197.1, 197.2, 197.3, 197.4, 197.5, 197.6, 197.7, 197.8, 198.0, 198.1, 198.2, 198.3, 198.4, 198.5, 198.6, 198.7, 198.81, 198.82, 198.89, 199.0, 199.1
AIDS/HIV	042, 043, 044, 044.9

Table S3. Association of Each Geriatric Condition with Total Hospital Cost and LOS

		Total Hospital Cost						LOS					
		Delirium		Dementia		Frailty		Delirium		Dementia		Frailty	
		Exp(Coef)	(95% CI)	Exp(Coef)	(95% CI)	Exp(Coef)	(95% CI)	IRR	(95% CI)	IRR	(95% CI)	IRR	(95% CI)
Geriatric condition		1.32	(1.25–1.38)	1.00	(0.96–1.04)	1.07	(1.03–1.12)	1.70	(1.60–1.81)	1.02	(0.95–1.10)	1.23	(1.15–1.31)
Age group	65–79	Reference						Reference					
	80–89	0.97	(0.95 - 1.00)	0.97	(0.95 - 1.00)	0.97	(0.95 - 1.00)	0.97	(0.93–1.02)	0.98	(0.93–1.02)	0.97	(0.92–1.01)
	>90	1.00	(0.96–1.03)	1.00	(0.96–1.03)	0.99	(0.96–1.03)	0.96	(0.90–1.02)	0.96	(0.90–1.02)	0.94	(0.89–1.00)
Female		1.03	(1.01 - 1.05)	1.03	(1.01–1.05)	1.03	(1.00–1.05)	1.07	(1.03–1.11)	1.07	(1.03–1.12)	1.07	(1.02–1.11)
Race	White	Reference						Reference					
	Black	1.00	(0.94–1.06)	1.01	(0.95–1.07)	1.01	(0.95–1.07)	1.11	(1.00–1.22)	1.11	(1.00–1.24)	1.11	(1.00–1.24)
	Hispanic	1.11	(1.04–1.19)	1.11	(1.05–1.19)	1.12	(1.05–1.19)	1.15	(1.04–1.27)	1.16	(1.05–1.29)	1.17	(1.06–1.30)
	Asian	1.19	(1.06–1.33)	1.20	(1.07–1.35)	1.20	(1.07–1.35)	1.14	(0.94–1.38)	1.17	(0.95–1.44)	1.16	(0.94–1.43)
	Native American	1.12	(0.95–1.31)	1.13	(0.92–1.38)	1.14	(0.93–1.39)	1.35	(0.87–2.08)	1.32	(0.83–2.08)	1.35	(0.85–2.14)
	Other	1.02	(0.97–1.08)	1.03	(0.97–1.09)	1.03	(0.97–1.09)	1.10	(1.00–1.20)	1.09	(1.00–1.20)	1.10	(1.00–1.21)
Insurance	Medicare/Medicaid	Reference						Reference					
	Private insurance	0.99	(0.95–1.04)	1.00	(0.95–1.05)	1.00	(0.95–1.05)	0.94	(0.86–1.02)	0.95	(0.87–1.04)	0.95	(0.87–1.05)
	Self-pay	1.27	(1.09–1.47)	1.29	(1.11–1.50)	1.29	(1.11–1.50)	1.15	(0.88–1.49)	1.17	(0.90–1.52)	1.18	(0.91–1.53)
	Other	1.03	(0.94–1.14)	1.02	(0.92–1.12)	1.02	(0.92–1.12)	0.85	(0.74–0.97)	0.82	(0.72–0.94)	0.82	(0.71–0.94)
Elective admission		0.84	(0.82–0.87)	0.84	(0.82–0.86)	0.84	(0.82–0.86)	0.58	(0.56–0.61)	0.58	(0.55–0.60)	0.58	(0.55–0.60)
Urban location		1.07	(1.04–1.10)	1.07	(1.04–1.10)	1.07	(1.04–1.10)	1.06	(1.01–1.11)	1.04	(0.99–1.10)	1.05	(0.99–1.10)
Atrial fibrillation		1.04	(1.02–1.06)	1.04	(1.02–1.07)	1.04	(1.02–1.07)	1.15	(1.11–1.19)	1.15	(1.11–1.20)	1.15	(1.11–1.20)
Cerebrovascular disease		1.04	(1.01–1.07)	1.05	(1.02–1.09)	1.05	(1.02–1.08)	1.08	(1.02–1.14)	1.11	(1.05–1.17)	1.11	(1.05–1.17)
Coronary arterial disease		0.93	(0.91–0.96)	0.92	(0.90–0.95)	0.93	(0.90–0.95)	0.90	(0.86–0.95)	0.89	(0.85–0.93)	0.89	(0.85–0.94)
Congestive heart failure		1.06	(1.03–1.09)	1.06	(1.04–1.09)	1.06	(1.03–1.09)	1.12	(1.07–1.17)	1.12	(1.07–1.17)	1.12	(1.07–1.17)
Hypertension		0.88	(0.85–0.91)	0.87	(0.84–0.90)	0.87	(0.85–0.90)	0.81	(0.77–0.86)	0.80	(0.76–0.84)	0.80	(0.76–0.84)
Chronic kidney disease		1.08	(1.06–1.11)	1.09	(1.06–1.11)	1.09	(1.06–1.11)	1.21	(1.17–1.26)	1.23	(1.18–1.28)	1.23	(1.18–1.28)
Chronic pulmonary obstructive disease		1.01	(0.98–1.03)	1.01	(0.98–1.03)	1.01	(0.98–1.03)	1.04	(0.99–1.08)	1.04	(0.99–1.08)	1.03	(0.98–1.07)
Peripheral arterial disease		1.04	(1.02–1.07)	1.04	(1.01–1.07)	1.04	(1.01–1.07)	0.99	(0.95–1.04)	0.99	(0.95–1.04)	0.99	(0.95–1.04)
Cancer		0.97	(0.95–1.00)	0.97	(0.94–0.99)	0.97	(0.94–0.99)	0.94	(0.90–0.98)	0.92	(0.88–0.97)	0.93	(0.89–0.97)
Diabetes		0.99	(0.97–1.01)	0.99	(0.96–1.01)	0.99	(0.96–1.01)	0.96	(0.92–1.00)	0.95	(0.92–0.99)	0.96	(0.92–1.00)
Liver disease		1.24	(1.16–1.31)	1.25	(1.18–1.33)	1.25	(1.18–1.33)	1.37	(1.25–1.50)	1.40	(1.27–1.55)	1.40	(1.27–1.55)
Other valvular disease		0.97	(0.95–1.00)	0.97	(0.94–1.00)	0.97	(0.94–1.00)	0.97	(0.93–1.02)	0.97	(0.93–1.02)	0.97	(0.92–1.02)

Abbreviations: CI, Confidence interval; Exp(Coef), exponential of coefficient; IRR, incidence rate ratio; LOS, length of stay.

Table S4. Association of Interaction Terms of Geriatric Conditions with Total Hospital Cost and LOS

		Exp(Coef)	(95% CI)	IRR	(95% CI)
Dementia		1.007	(0.959–1.058)	0.997	(0.920–1.081)
Frailty		1.049	(1.009–1.090)	1.202	(1.121–1.288)
Delirium		1.329	(1.257–1.404)	1.745	(1.627–1.871)
Dementia # Frailty		1.017	(0.897–1.153)	1.001	(0.780–1.284)
Dementia # Delirium		0.829	(0.729–0.942)	0.785	(0.643–0.959)
Frailty # Delirium		1.092	(0.942–1.265)	0.952	(0.789–1.149)
Dementia # Frailty # Delirium		0.822	(0.643–1.052)	1.044	(0.705–1.546)
Age group	65–79	Reference			
	80–89	0.972	(0.948–0.998)	0.969	(0.927–1.013)
	>90	0.993	(0.960–1.028)	0.949	(0.896–1.005)
Female		1.029	(1.006–1.052)	1.063	(1.021–1.106)
Race	White	Reference			
	Black	0.996	(0.943–1.053)	1.103	(0.999–1.217)
	Hispanic	1.114	(1.046–1.188)	1.158	(1.050–1.276)
	Asian	1.187	(1.060–1.330)	1.127	(0.929–1.367)
	Native American	1.122	(0.955–1.317)	1.369	(0.885–2.118)
	Other	1.025	(0.970–1.083)	1.102	(1.008–1.205)
Insurance	Medicare/Medicaid	Reference			
	Private insurance	0.998	(0.951–1.046)	0.948	(0.870–1.034)
	Self-pay	1.281	(1.103–1.488)	1.161	(0.896–1.504)
	Other	1.033	(0.935–1.140)	0.840	(0.731–0.965)
Elective admission		0.843	(0.820–0.866)	0.581	(0.558–0.606)
Urban location		1.070	(1.042–1.099)	1.058	(1.008–1.112)
Atrial fibrillation		1.039	(1.017–1.061)	1.146	(1.104–1.189)
Cerebrovascular disease		1.040	(1.008–1.074)	1.081	(1.025–1.140)
Coronary arterial disease		0.933	(0.909–0.958)	0.906	(0.864–0.950)
Congestive heart failure		1.059	(1.033–1.085)	1.117	(1.070–1.166)
Hypertension		0.882	(0.854–0.911)	0.817	(0.777–0.860)
Chronic kidney disease		1.082	(1.057–1.107)	1.208	(1.161–1.257)
Chronic pulmonary obstructive disease		1.007	(0.983–1.032)	1.028	(0.986–1.071)
Peripheral arterial disease		1.040	(1.014–1.067)	0.993	(0.951–1.036)
Cancer		0.973	(0.950–0.997)	0.938	(0.898–0.980)
Diabetes		0.989	(0.967–1.011)	0.961	(0.926–0.998)
Liver disease		1.236	(1.163–1.312)	1.367	(1.246–1.501)
Other valvular disease		0.972	(0.945–0.999)	0.971	(0.924–1.021)

Abbreviations: CI, confidence interval; Exp(Coef), exponential of coefficient; IRR, incidence rate ratio; LOS, length of stay.
